# Integration naturwissenschaftlicher und medizinischer Grundlagen im Hamburger Modellstudiengang Zahnmedizin iMED DENT

**DOI:** 10.1007/s00103-023-03795-0

**Published:** 2023-11-14

**Authors:** Roland Bender, Natascha Bruhn, Sophie Eisenbarth, Rüdiger Lemke, Chiara-Fabienne Pantke, Wolfgang Hampe, Alexander Peter Schwoerer

**Affiliations:** 1 Institut für Neuroanatomie, Universitätsklinikum Hamburg-Eppendorf, Hamburg, Deutschland; 2 Poliklinik für Kieferorthopädie, Universitätsklinikum Hamburg-Eppendorf, Hamburg, Deutschland; 3 Prodekanat für Lehre, Universitätsklinikum Hamburg-Eppendorf, Hamburg, Deutschland; 4 Poliklinik für Zahnärztliche Prothetik, Universitätsklinikum Hamburg-Eppendorf, Hamburg, Deutschland; 5 Poliklinik für Parodontologie, Präventive Zahnmedizin und Zahnerhaltung, Universitätsklinikum Hamburg-Eppendorf, Hamburg, Deutschland; 6 Institut für Biochemie und Molekulare Zellbiologie, Universitätsklinikum Hamburg-Eppendorf, Hamburg, Deutschland; 7 Institut für Zelluläre und Integrative Physiologie, Universitätsklinikum Hamburg-Eppendorf, Martinistr. 52, 20246 Hamburg, Deutschland

**Keywords:** Zahnmedizin, Integration, Naturwissenschaften, Grundlagenfächer, Vorklinik, Integrated dentistry program, Integration, Natural sciences, Medical basics, Preclinical

## Abstract

Im Oktober 2019 startete in Hamburg mit dem Studiengang iMED DENT erstmals in Deutschland ein integrierter Modellstudiengang der Zahnmedizin. Mit diesem Studiengang werden Lehrkonzepte wie integrierte Lehre, früher Patient:innenkontakt und früher Wissenschaftsbezug, die in den vergangenen Jahren in der Humanmedizin erfolgreich getestet wurden, auf die Zahnmedizin übertragen. Der Studienabschnitt „Normalfunktion“ im ersten Jahr des Studiengangs orientiert sich im Wesentlichen am gesunden Menschen. Ein wesentliches Augenmerk wurde in diesem Abschnitt auf die Integration der naturwissenschaftlichen und medizinischen Grundlagen der Zahnmedizin sowie auf einen frühen praktischen Bezug gelegt.

Aus den Erfahrungen der ersten 4 Kohorten lassen sich erste Rückschlüsse über diesen Studienabschnitt ziehen. Seine modulare Struktur sorgt im Allgemeinen für eine Straffung der Lehrinhalte, das Angebot der integrierten Lehre wird insgesamt sehr gut angenommen. So wird beispielsweise die Präsentation naturwissenschaftlicher Grundlagen im Kontext von zahnmedizinischer Relevanz von den Studierenden des Modellstudienganges deutlich positiver bewertet als die analogen Lehrangebote von Studierenden des Regelstudienganges. In ähnlicher Weise werden der integrierte Unterricht zahnmedizinischer und medizinischer Grundlagenfächer und die frühe Einbeziehung klinischer Aspekte in den Lehrevaluationen geschätzt. Die Integration naturwissenschaftlicher und medizinischer Grundlagen findet ihre Grenzen beispielsweise in der starken Praxisorientierung des Zahnmedizinstudiums, die häufig nur wenige unmittelbare Anknüpfungspunkte für nicht-zahnmedizinische Inhalte bietet. Eine iterative Abstimmung der beteiligten Fächer lässt hier eine weiter zunehmende Verzahnung der Lehrinhalte erwarten.

## Abstract (en)

In October 2019, an integrated dentistry program (iMED DENT) was implemented at the University of Hamburg and was the first of its kind in Germany. This model curriculum builds on didactic concepts that have been applied successfully for many years in curricula for human medicine, including interdisciplinary teaching, early clinical experience, and scientific education. The first year focuses on the healthy situation (“normal function”) and aims to integrate the natural sciences (biology, chemistry, physics) and the basic medical subjects (anatomy, biochemistry, physiology, medical terminology) in the context of dental health. Further, basic practical and clinical tasks are assigned to the students during the first year.

From the experience of the first four cohorts, initial conclusions can be drawn about this stage of study. Generally, its modular structure results in a condensation of learning content, which students judge as demanding. However, its interdisciplinary approach is well accepted. For instance, presenting the basics of the natural sciences in the context of their dental relevance is much better evaluated in the new compared to the previous curriculum, in which this content was taught without specific references to dental health. Teaching the basics of medicine within clinical context and the inclusion of early clinical practice are similarly appreciated. Presently, the interdisciplinary approach is limited by the focus on practical competencies of the dentistry curriculum, as some practical courses offer only few opportunities for other disciplines to interconnect their teaching. The continuous evaluation of the curriculum and exchange of experiences between the disciplines will further improve the integrative concept of the curriculum.

## Einleitung

Im Oktober 2019 startete am Universitätsklinikum Hamburg-Eppendorf mit dem Studiengang iMED DENT erstmals in Deutschland ein integrierter Modellstudiengang der Zahnmedizin. Kernziele bei der Entwicklung stellten die Vernetzung aller beteiligten Fächer in integrierten Modulen sowie ein enger klinischer und wissenschaftlicher Bezug von Studienbeginn an dar (siehe auch Beitrag von Guse et al. in diesem Themenheft). Dies betraf auch die Vermittlung naturwissenschaftlicher Grundlagen in Biologie, Chemie und Physik, die zuvor von der Fakultät für Mathematik, Informatik und Naturwissenschaften (MIN) der Universität gelehrt wurden. Diese außerhalb der medizinischen Fakultät gelagerte Lehre wurde von den Studierenden regelmäßig aufgrund eines geringen zahnmedizinischen Fachbezugs als „unbefriedigend“ angesehen. Darüber hinaus bestand der Konsens, die Lehre der medizinischen Grundlagenfächer (Anatomie, Biochemie, Physiologie und medizinische Terminologie) durch eine engere Anbindung an die zahnmedizinischen Fächer und durch eine stärkere Fokussierung auf zahnmedizinisch relevante Lehrinhalte zu stärken.

Folglich wurde bei der Studiengangentwicklung auf die Integration naturwissenschaftlicher und medizinischer Grundlagen in einen zahnmedizinisch relevanten Kontext fokussiert (Studienabschnitt „Normalfunktion“ im ersten Studienjahr). Zentral war somit zunächst die Formulierung des für die spätere praktische und wissenschaftliche zahnmedizinische Tätigkeit notwendigen Wissens sowie der relevanten Kompetenzen und Fertigkeiten. Die inhaltliche Abstimmung erfolgte dabei unter Berücksichtigung des Nationalen Kompetenzbasierten Lernzielkatalogs Zahnmedizin (NKLZ). Ausgehend von diesen zahnmedizinischen Inhalten wurden Schnittstellen mit anderen Fächern identifiziert. Die vernetzten Lehrveranstaltungen wurden dann in einem möglichst geringen zeitlichen Abstand zueinander im Stundenplan angeordnet. Schließlich wurden die naturwissenschaftlichen Lehrinhalte den Fächern zugeordnet, die inhaltlich auf diesen aufbauen, und dem jeweiligen Kontext entsprechend im Stundenplan verortet.

Das Curriculum ist modular organisiert. Für die Module wurden übergeordnete Modulthemen und Unterthemen für einzelne Modulabschnitte erarbeitet, die als Leitschienen für die zu integrierenden Lehrinhalte dienten. Nach Beginn des Modellstudiengangs erfolgte auf Grundlage der studentischen Lehrevaluation und der sich stetig verbessernden Abstimmung der beteiligten Fächer eine sukzessiv zunehmend engere Verzahnung der Inhalte.

Der Lernerfolg eines jeden Moduls wird mittels interdisziplinärer Modulprüfungen überprüft. Ein Modul gilt als bestanden, wenn über alle Fächer hinweg mindestens 60 % der Maximalpunktzahl erreicht werden. Zusätzlich gilt diese Grenze auch für jeden einzelnen praktischen Prüfungsanteil der zahnmedizinischen Kurse. Die Fächer Physik, Chemie und Biologie werden jeweils im Rahmen mehrerer Module geprüft und müssen – jedes Fach für sich – ebenfalls mit mindestens 60 % bestanden werden. Darüber hinaus werden am Ende des ersten Studienjahres die manuell-zahnärztlichen Fertigkeiten in einer Fortschrittsprüfung (MFP) getestet, deren Bestehen Voraussetzung für das Fortschreiten ins zweite Studienjahr ist.

Im Folgenden werden die zentralen Elemente des integrierten Unterrichts im ersten Studienjahr in der Übersicht vorgestellt und die Prüfungsformate und begleitenden Studienangebote erläutert. Eine detaillierte Darstellung des integrativen Konzepts erfolgt dabei an einem ausgewählten Modul. Darüber hinaus werden wesentliche Herausforderungen und Limitationen aus unserer Sicht dargestellt.

## Die Module der „Normalfunktion“

Im ersten Studienjahr erfolgt die Vermittlung naturwissenschaftlicher Grundlagen und zahnmedizinischer Basiskenntnisse („Normalfunktion“) interdisziplinär in 4 Modulen von je 7 Wochen (siehe auch Beitrag von Guse et al. in diesem Themenheft). Die naturwissenschaftlichen (Biologie, Chemie, Physik) und medizinischen Grundlagen (Anatomie, Physiologie, Biochemie, medizinische Soziologie, medizinische Psychologie, medizinische Terminologie) werden darin mit der theoretisch-praktischen Ausbildung in den zahnmedizinischen Fächern verknüpft (Werkstoffkunde, Kieferorthopädie, zahnärztliche Prothetik und Zahnerhaltung). Durch das Training feinmotorischer Basisfertigkeiten werden die Studierenden frühzeitig an die klinische Tätigkeit herangeführt. Zusätzlich sind longitudinale Stränge zu Grundkompetenzen in wissenschaftlichem Arbeiten und Kommunikation implementiert. Die Module der „Normalfunktion“ werden im Folgenden näher skizziert; eine Übersicht zu den Schwerpunkten findet sich in Tab. 1.

**Table 1:** 

	Modul A: Naturwissenschaftliche und zahnmedizinische Grundlagen	Modul B1: Gewebe und Funktionen des oralen Systems	Modul B2: Präklinisches Training und systemische Aspekte	Modul B3: Form, Funktion, Forschung
Anatomie	Histologie (Grundgewebe), Zahn, Zahnhalteapparat, Epithelien der Mundhöhle	Kiefergelenk, Kaumuskulatur, Schädel, orofaziales System	Anatomie des Halses und des Thorax (v. a. Kehlkopf, Herz, Lunge)	Anatomie des Abdomens (v. a. Darmtrakt, Harnwege)
Biochemie	–	Proteinbiosynthese, Enzyme	–	–
Biologie	Aufbau der Zelle, Gene, Evolution	Humangenetik	Mikrobiologie (Bakterien, Viren)	–
Chemie	Elemente, Moleküle, Reaktionen, Säuren und Basen, Puffer	Metalle, Thermodynamik, Kinetik, Katalyse, Polymere	–	–
Kieferorthopädie	–	Orthopädie, Orthodontie, Kieferentwicklung, Zahnwechsel, Gebissanomalien, Drahtbiegeübungen	Kieferorthopädische Anamnese, Herstellung eines Schaumodells	–
Medizinische Soziologie	–	–	Soziale Faktoren der Mundgesundheit	–
Medizinische Terminologie	–	Medizinische Fachbegriffe des oralen Systems	–	–
Osteologie	–	Knochenbiologie	–	–
Physik	Ionisierende Strahlung, Grundlagen des Röntgens	Biomechanik	Biomechanik, Elektrizitätslehre	Drücke, Strömungen, Wellen, Optik
Physiologie	–	–	Zell‑, Nerven- und Muskelphysiologie, Atmung	Herz-Kreislauf-System, Gastrointestinaltrakt, Säure-Basen-Haushalt, Nierenfunktion
Werkstoffkunde	Wachse, Gipse	Metalle, Polymere, Komposite, Schleifkörper	Modellwerkstoffe, Abformmassen	CAD/CAM-Technologie
Zahnärztliche Prothetik	Zahnentwicklung, Zahnaufbau, spezielle Zahnmerkmale, Zahnhalteapparat, Herstellung von Wachszähnen	Orofaziales System, Okklusion, Kiefergelenk, Simulation von Kieferbewegung, Registrierverfahren, orofaziale Ästhetik	Funktionelle Untersuchung, Kieferrelationsbestimmung, Abformung, Modellherstellung und -bearbeitung, Herstellung einer Michigan-Schiene	Grundlagen CAD/CAM, Okklusionskonzepte, Zahnaufstellung, dentofaziale Ästhetik, anatomoforme Kauflächenpräparation
Zahnerhaltung	–	Präparationsübungen	Berufsfelderkundung (Ergonomie und Befundaufnahme), Basishygiene	–

*CAD* Computer-aided design, *CAM* Computer-aided manufacturing

### Modul A – Naturwissenschaftliche und zahnmedizinische Grundlagen

Dieses Modul ist thematisch in 2 Abschnitte untergliedert. In „Der Zahn“ (Woche 1–4) stehen die Entwicklung, der anatomische Aufbau und die Zusammensetzung von Zähnen im Mittelpunkt. Aufeinander Bezug nehmend werden hier von den beteiligten Fächern die Entwicklung und Histologie der Zähne (Biologie, Anatomie, zahnärztliche Prothetik), ihre chemischen Komponenten (Chemie) sowie der Aufbau und die Charakteristika einzelner Zähne (zahnärztliche Prothetik) behandelt. Analog werden im nachfolgenden Abschnitt „Der Zahn und seine Umgebung“ (Woche 5–7) der Aufbau und die Funktionen des Zahnhalteapparates und der Mundschleimhaut abgestimmt aus den Perspektiven der zahnärztlichen Prothetik, der Anatomie und der Chemie (z. B. Puffer im Speichel) besprochen. Darüber hinaus erlernen die Studierenden im ersten praktischen Kurs der zahnärztlichen Prothetik die manuellen Fertigkeiten, Zähne anatomisch korrekt abzubilden, und werden an die späteren zahnärztlichen Tätigkeiten und die täglichen Werkstoffe herangeführt. Ergänzend erfolgt eine Einweisung in die biologische und diagnostische Bedeutung von ionisierender Strahlung (Physik/zahnärztliches Röntgen).

### Modul B1 – Gewebe und Funktionen des oralen Systems

In diesem Modul werden in den praktischen zahnmedizinischen Kursen durch die Herstellung von Drahtbiegefiguren in der Kieferorthopädie und durch Präparationsübungen der Zahnerhaltung und zahnärztlichen Prothetik die feinmotorischen Fertigkeiten geschult. Anhand dieses Moduls sollen die Integration und zeitliche Koordination der naturwissenschaftlich-medizinischen Fächer exemplarisch näher ausgeführt werden (Tab. 2).

**Table 2:** 

Modul B1: Gewebe und Funktionen des oralen Systems							
	Gene und Proteine im Mund		Zahn- und Kieferbewegung		Intraoral: Mund und Rachen	Extraoral: Das Gesicht	
	1. Woche	2. Woche	3. Woche	4. Woche	5. Woche	6. Woche	7. Woche
Zahnerhaltung	–	–	Zahnerhaltung und Prävention Präparation an Klötzchen		Präparation an Klötzchen	Präparation an Klötzchen	
Prothetik, Werkstoffkunde	Speichel, Metalle	Okklusion, Schleifkörper	Kiefergelenk, Polymere	Simulation Kieferbewegung, Komposite	Registrierverfahren	Orofaziale Ästhetik	Repetitorium
Anatomie	–	–	Gesichts- und Schädel-Entwicklung, Schädel	Kiefergelenk, Kieferbewegung, Kaumuskulatur	Mund, Zunge, Rachen, Speicheldrüsen	Mimische Muskulatur, Blutversorgung Kopf	–
Med. Terminologie	–	–	–	Schädel	Mund, Gesicht	–	Modulklausur
Kieferorthopädie	Orthopädie + Orthodontie, Kieferentwicklung	Befunde, Diagnostik (Prakt.: Drahtbiegen)	Zahnwechsel, Gebissanomalien (Prakt.: Drahtbiegen)		Gebissanomalien	Gebissanomalien	
Crashkurse	Chemie (Metalle, Amino-säuren), Biologie (Genetik)	Physik (Mechanik)	Chemie (Polymere)	–	–	–	–
Physik (Kieferorthopädie, Radiologie)	–	Biomechanik	Biomechanik, Zahnbewegung		Biomechanik	–	Modulklausur
Osteologie	–	–	–	Knochenbiologie	–	–	–
Chemie	Metalle	Thermodynamik, Kinetik, Katalyse	Polymere	–	–	–	Mündliche Prüfung
Biologie (Humangenetik)	Genom des Menschen	Mutationen: klinische Ausprägung und Vererbung	–	–	–	–	Modulklausur
Biochemie	Proteinbiosynthese im Mund	Enzyme (u. a. im Speichel), Blutgerinnung	–	–	–	–	Mündliche Prüfung

In den ersten 2 Modulwochen werden unter dem Thema „Gene und Proteine im Mund“ die molekularen Grundlagen z. B. von Genetik, Proteinbiosynthese und Enzymkatalyse aus den Bereichen der Chemie, Biologie (Humangenetik) und Biochemie integriert, nachdem die schulischen Grundlagen in Crashkursen wiederholt wurden. Im Kurs der Kieferorthopädie werden parallel Drähte gebogen, dazu passend werden in der Werkstoffkunde und der Chemie Metalle und in der Physik die Biomechanik behandelt.

Im zweiten Abschnitt „Zahn- und Kieferbewegung“ werden in der dritten und vierten Modulwoche Aufbau und Funktion der Kaumuskulatur, das Kiefergelenk und Aspekte der Kieferentwicklung (z. B. Zahnwechsel) aus Sicht der zahnärztlichen Prothetik, der Kieferorthopädie und der Anatomie unterrichtet. Die physikalischen Grundlagen zur Biomechanik und speziell der Zahnbewegung werden vertiefend durch die Lehrenden der Kieferorthopädie unterrichtet und durch Ausführungen zur Knochenbiologie von der Osteologie ergänzt.

Im dritten Abschnitt „Intraoral: Mund und Rachen“ wird die Thematik des oralen Systems auf Bereiche der Mundhöhle erweitert. Ergänzend zum Thema Speichel, das im ersten Abschnitt den Fächern zahnärztliche Prothetik und Biochemie eingeführt wird, werden im Fach Anatomie die Speicheldrüsen und auch weitere Strukturen im Mund behandelt. Wie im zweiten Abschnitt wird die Verwendung von Fachbegriffen parallel im Fach medizinische Terminologie erläutert.

Im letzten Abschnitt „Extraoral: Das Gesicht“ unterrichten die Lehrenden der zahnärztlichen Prothetik die „orofaziale Ästhetik“ und passend dazu im Fach Anatomie die „mimische Muskulatur“. Das Thema „Blut“ aus dem ersten Abschnitt wird ergänzt durch Anatomieveranstaltungen zur Blutversorgung des Kopfes. Gegen Ende des Moduls wird in einem Teamteaching zum Thema „Röntgenanatomie“ von Vertreter:innen des zahnärztlichen Röntgens und der Anatomie gemeinsam die Bedeutung von Schädelstrukturen im Kieferbereich in bildgebenden Verfahren erläutert. In der siebenten und letzten Woche liegen die fächerübergreifende Modulabschlussklausur und eine strukturierte mündliche Prüfung der Chemie und Biochemie, die die modulbegleitenden Prüfungsanteile z. B. in den zahnmedizinischen Kursen ergänzen.

### Modul B2 – Präklinisches Training und systemische Aspekte

Dieses Modul fokussiert auf die Schwerpunkte „Der erste Patient:innenkontakt“ sowie erweiterte Funktionen des orofazialen Systems („Schlucken, Sprechen, Atmen“). Praktische Kurse der zahnärztlichen Prothetik und Kieferorthopädie erstrecken sich über das gesamte Modul.

Im Rahmen eines „Teamteachings“ (zahnärztliche Prothetik/Kieferorthopädie) führen die Studierenden in der Zahnklinik gegenseitig eine zahnärztliche Abdrucknahme mit Alginat und die Kieferrelationsbestimmung durch. Hierbei werden die Besonderheiten einer kieferorthopädischen bzw. prothetischen Abformung und der klinische Ablauf im Behandlungsalltag nahegebracht. Parallel erfolgt für den späteren synoptischen Behandlungskurs die Schulung zum Hygieneverhalten. Im Labor stellen die Studierenden 2 Arbeitsmodelle aus Superhartgips und Hartgips her. Im Kurs der zahnärztlichen Prothetik lernen sie die Übertragung der Kieferrelationsbestimmung in den Artikulator sowie die Funktion und Anfertigung einer Michigan-Schiene. Nach der Fertigstellung erfolgen in der Zahnklinik die Einprobe und Anpassung der Schiene an einem/r Patient:in bzw. Kommiliton:in sowie die Überprüfung ihrer Funktionalität.

Im Kurs der Kieferorthopädie stellen Studierende aneinander ein kieferorthopädisches Schaumodell her, wobei auf anatomische Strukturen, die in jedem Fall erhalten werden sollen, Wert gelegt wird. Diese praktischen Aspekte des Patient:innenkontakts werden im Rahmen des integrativen Unterrichts durch gegenseitige Anamneseerhebungen und gegenseitige zahnärztliche Befundaufnahme und Ergonomie am Arbeitsplatz durch das Fach Zahnerhaltung unterstützt. Hier beginnt mit Seminaren und praktischen Übungen zur Gesprächsführung auch der longitudinale Strang „Psychosoziale Kompetenzen und Kommunikation“ (siehe Abschnitt „Longitudinale Stränge“ unten und Beitrag von Guse et al. in diesem Themenheft).

Im zweiten Themenkomplex des Moduls („Schlucken, Sprechen, Atmen“) lernen die Studierenden parallel zu den weiterlaufenden zahnmedizinischen Kursen (s. oben) durch anatomische Präparierübungen die Anatomie des Halses (u. a. Kehlkopf) und des Thorax (Lunge, Herz) kennen. Funktionell werden sie gleichzeitig in die Grundlagen der Muskel- und Nervenphysiologie sowie die Physiologie der Atmung eingewiesen. Aus den naturwissenschaftlichen Grundlagen werden hierbei Inhalte der Physik (Elektrizitätslehre) integriert. Schließlich finden sich ergänzende Lehrveranstaltungen zur Mikrobiologie, Biomechanik und Strahlenphysik sowie zu sozialen Faktoren der Mundgesundheit, die es im Beruf zu berücksichtigen gilt (medizinische Soziologie).

### Modul B3 – Form, Funktion, Forschung

Dieses Modul beschließt den Studienabschnitt „Normalfunktion“ mit systemischen Aspekten der Form und (Normal‑)Funktion im Kontext des gesamten Organismus. Dabei werden auch kontinuierlich pathophysiologische Überlegungen thematisiert, um auf Patient:innen mit zahnmedizinisch relevanten (z. B. internistischen) Vorerkrankungen vorzubereiten. Schwerpunkte stellen hierbei neben dem Herz-Kreislauf-System, die Funktionen des Gastrointestinaltrakts, der Säure-Basen-Haushalt, der Wasserhaushalt und die Nierenfunktion dar.

Die entsprechenden Lehrinhalte der Anatomie und der Physiologie wurden dazu zeitlich und inhaltlich eng aufeinander abgestimmt. Naturwissenschaftliche Grundlagen werden beispielsweise aus der Physik, z. B. zur Strömungslehre und Wellenphysik, unterrichtet. Die zahnärztliche Prothetik adressiert die Modulthemen durch subtraktive und additive Kauflächengestaltung, bei denen auch ästhetische Aspekte im Mittelpunkt stehen („Form“), sowie durch digitale und optische Abformungsprozesse.

Schließlich beginnt für die Studierenden in diesem Modul der Longitudinalstrang zum „Wissenschaftlichen Arbeiten“ („Forschung“, siehe Abschnitt „Longitudinale Stränge“). Die Studierenden gewinnen hier zunächst Einblicke in die wissenschaftlichen Schwerpunkte der zahnmedizinischen Fächer an der medizinischen Fakultät. Darüber hinaus werden Wissenschaftsethik und gute wissenschaftliche Praxis thematisiert. Die Lehre erfolgt hierbei in Tandems von forschenden Wissenschaftler:innen aus den Bereichen der Statistik, Biometrie und den zahnmedizinischen Fächern.

### Studentische Evaluation

**Figure 1:**
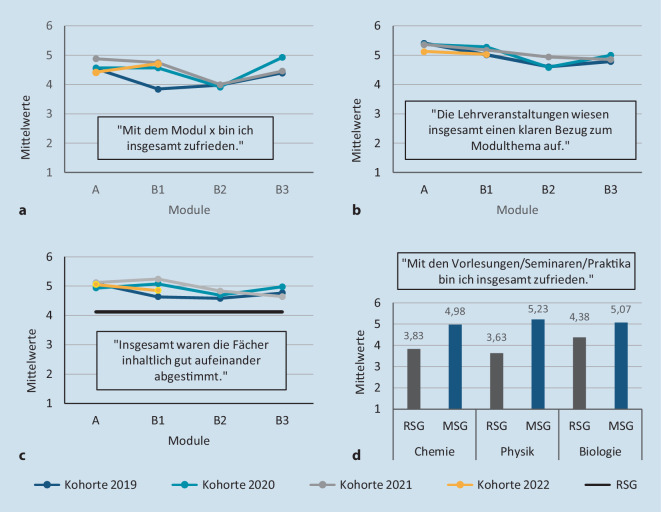

Im Rahmen der studentischen Evaluation werden systematisch verschiedene Aspekte zum Studiengang von den Studierenden bewertet. Nach dem Start des Modellstudiengangs im Jahr 2019 befindet sich aktuell der vierte Jahrgang im Studienabschnitt „Normalfunktion“. Trotz der eingeschränkten Rahmenbedingungen aufgrund der Corona-Pandemie wurde die integrierte Vermittlung von naturwissenschaftlichen und medizinischen Grundlagen im zahnmedizinischen Kontext von den Studierenden der ersten Jahrgänge sehr gut angenommen. So erhielten in der studentischen Lehrevaluation die Fragen zur Zufriedenheit mit den Modulen A bis B3 (Abb. 1a) und zum Bezug der Lehrveranstaltungen zum Modulthema (Abb. 1b) sehr gute Zustimmungswerte. Im Vergleich zum vorherigen Regelstudiengang zeigt sich in allen Modulen der Erfolg der Integration durch eine substanziell höhere Bewertung bei der Abstimmung der Fächer (Abb. 1c) und bei der Zufriedenheit mit den naturwissenschaftlichen Grundlagenfächern (Abb. 1d).

## Longitudinale Stränge

### „Psychosoziale Kompetenzen und Kommunikation“ und „Wissenschaftliches Arbeiten“

Neben der Vermittlung naturwissenschaftlicher und medizinischer Grundlagen im zahnmedizinischen Kontext beinhaltet das erste Studienjahr Lehrveranstaltungen der Stränge „Psychosoziale Kompetenzen und Kommunikation“ und „Wissenschaftliches Arbeiten“, die über das Studium hinweg longitudinal durchgeführt werden (siehe auch Beiträge von Guse et al. und Lemke et al. in diesem Themenheft). Diese Stränge wurden bei der Studiengangentwicklung, ausgehend vom zahnärztlichen Bedarf, zeitgleich zur Entwicklung der weiteren Inhalte definiert. Bei der Entwicklung der Stränge flossen Erfahrungen zu analogen Konzepten aus dem integrierten Modellstudiengang Medizin am Standort ein, die auf die speziellen Anforderungen und Rahmenbedingungen des zahnmedizinischen Studiums angepasst wurden [1, 2].

Der Longitudinalstrang „Psychosoziale Kompetenzen und Kommunikation“ beginnt im Modul B2 mit einer Reihe von Seminaren (medizinische Psychologie), in denen zentrale Aspekte der Gesprächsführung mit Patient:innen behandelt und erste Gesprächstechniken eingeübt werden. Der Longitudinalstrang „Wissenschaftliches Arbeiten“ findet erstmalig im Modul B3 statt. Beide Lernschwerpunkte sind neu im Modellstudiengang iMED DENT und waren früher in der zahnmedizinischen Ausbildung nicht oder nur marginal repräsentiert. Die Lehrveranstaltungen werden in gemeinsamer Verantwortung der beteiligten zahnmedizinischen und nicht-zahnmedizinischen Fächer durchgeführt. Trotz der Einschränkungen durch die Corona-Pandemie in den ersten Kohorten kann für den ersten Studienabschnitt bereits eine sehr positive Bilanz zu den integrativen Strängen gezogen werden. Beispielsweise zeigen die Erfahrungen mit den ersten abgeschlossenen Studienarbeiten, dass die frühe Integration des „Wissenschaftlichen Arbeitens“ bereits im ersten Studienjahr zu einer intensiveren Auseinandersetzung mit der Wissenschaft in der Zahnmedizin führt als im vorherigen Regelstudiengang am Standort.

### Früher Patient:innenkontakt

Das Studium der Zahnmedizin muss die Studierenden befähigen, direkt nach dem Staatsexamen selbstständig die Behandlung von Patient:innen planen und durchführen zu können. Ein besonderes Ziel bei der Entwicklung des integrierten Curriculums lag daher auf der Vermittlung von praktischen Kompetenzen in einem vermehrten und frühen Patient:innenkontakt während des Studiums (siehe auch Beitrag von Guse et al. in diesem Themenheft).

Bereits im ersten Modul des zweiten Semesters (Modul B2) erheben die Studierenden die erste Anamnese, nehmen einen Befund auf und führen eine Abdrucknahme sowie eine Kieferrelationsbestimmung im Rahmen des prothetisch-kieferorthopädischen Teamteachings durch. Das frühzeitige Heranführen an die zahnärztliche Tätigkeit soll den Studierenden den zahnärztlichen Alltag näherbringen und sie in ihrem Berufswunsch bestärken. Des Weiteren gilt das Heranführen an die Behandlungseinheit und die Hygienekonzepte in der Zahnklinik als Vorbereitung für die Stuhlassistenz höherer Semester und den synoptischen Behandlungskurs. Dieses Konzept wird von den Studierenden in den bisherigen Durchläufen des Studienganges – trotz erschwerter Bedingungen in den Corona-Jahren – sehr positiv angenommen.

## Flankierende Maßnahmen

### Crashkurse

Naturwissenschaftliche Grundkenntnisse sind eine wichtige Voraussetzung für das Studium der Zahnmedizin, Studierende bringen diesbezüglich jedoch sehr unterschiedliche Vorkenntnisse mit. Ausgehend vom erfolgreichen Angebot aus dem Modellstudiengang Medizin (iMED) wurden integrierte Crashkurse zur Vermittlung bzw. Wiederholung zahnmedizinisch relevanter Grundkenntnisse in Biologie, Chemie und Physik entwickelt [3, 4]. Diese Crashkurse bieten thematisch am Curriculum ausgerichtete, begleitende Lehre in zeitlicher Nähe zu den Pflichtveranstaltungen (Tab. 2). Die 90-minütigen Crashkurse werden in Gruppen von ca. 20 Studierenden abgehalten, was ein interaktives, an den Bedürfnissen der Lerngruppe orientiertes Erarbeiten der Lernziele ermöglicht sowie Zeit und Raum für individuelle Fragen bietet.

Verteilt über den Studienabschnitt „Normalfunktion“ werden insgesamt 18 Crashkurse mit Bezug zum Curriculum angeboten. Schwerpunkte stellen hier in der Chemie/Biologie die Lehre zu Elementen, Molekülen, Säuren/Basen, anorganischer und organischer Chemie, Aminosäuren, Proteinen, Kohlenhydraten und Lipiden dar. Aus der Physik werden Strahlungen, Mechanik, Elektrizitätslehre, Drücke und Strömungen sowie Optik und Akustik thematisiert. Für jedes Thema werden parallel Kurse angeboten, die sich auf der Grundlage einer Selbsteinschätzung der Studierenden an deren unterschiedlichen Vorkenntnissen orientieren, um möglichst homogene Lerngruppen zu bilden. Die Teilnahme an den Kursen ist freiwillig, dennoch werden die Kurse von bis zu 80 % der Studierenden wahrgenommen.

Im Rahmen der Lehrevaluation wurden die Crashkurse von den Studierenden als sehr gut bewertet. Besonders mit den homogenen Lerngruppen zeigen sich die Studierenden sehr zufrieden.

### Studierendenauswahl

Studienprobleme bis hin zum Studienabbruch resultierten im Regelstudiengang Zahnmedizin zum Teil aus fehlendem manuellen Geschick und aus unzureichender schulischer Vorbildung in den Naturwissenschaften. Um hier gute Voraussetzungen bei den Studierenden zu erreichen, werden in der Studierendenauswahl der Drahtbiegetest HAM-Man und der Naturwissenschaftstest HAM-Nat hoch gewichtet, die einerseits die Leistung in den praktischen Kursen der zahnmedizinischen Fächer und zum anderen die theoretischen Studienleistungen in den ersten Studienjahren vorhersagen [5–11]. Motivierte Studienbewerber:innen bereiten sich intensiv auf diese Tests und somit auch auf das Studium vor. Zusätzlich werden soziale Fähigkeiten der Bewerber:innen, die z. B. für die Lehrveranstaltungen zur Kommunikation benötigt werden, im Situational Judgement Test (HAM-SJT) geprüft.

## Prüfungen

Im Studienabschnitt „Normalfunktion“ werden von den Studierenden 4 Modulprüfungen absolviert, die sich aus der Gesamtheit der für das Modul vorgesehenen modulbegleitenden Teilleistungen (mündliche und schriftliche Prüfungen in den Grundlagenfächern, praktische Arbeiten in den zahnmedizinischen Fächern) und einer Modulabschlussprüfung (fächerübergreifende Multiple-Choice-Klausur, strukturierte mündliche Prüfungen) zusammensetzen. Insgesamt können pro Modul 100 Punkte erreicht werden, wobei 30–50 % der Punkte von den zahnmedizinischen Fächern – überwiegend für praktische Leistungen – vergeben werden. Durch die hohe Gewichtung der praktischen Anteile soll die Ausbildung feinmotorischer Fähigkeiten früh intensiviert und den Studierenden eine frühzeitige Einschätzung über ihre persönliche Eignung zur Zahnmedizin ermöglicht werden. Zum Bestehen eines Moduls müssen mindestens 60 % der Gesamtmodulpunkte und mindestens 60 % der praktisch-zahnmedizinischen Prüfungsinhalte erfolgreich abgeleistet werden.

**Figure 2:**
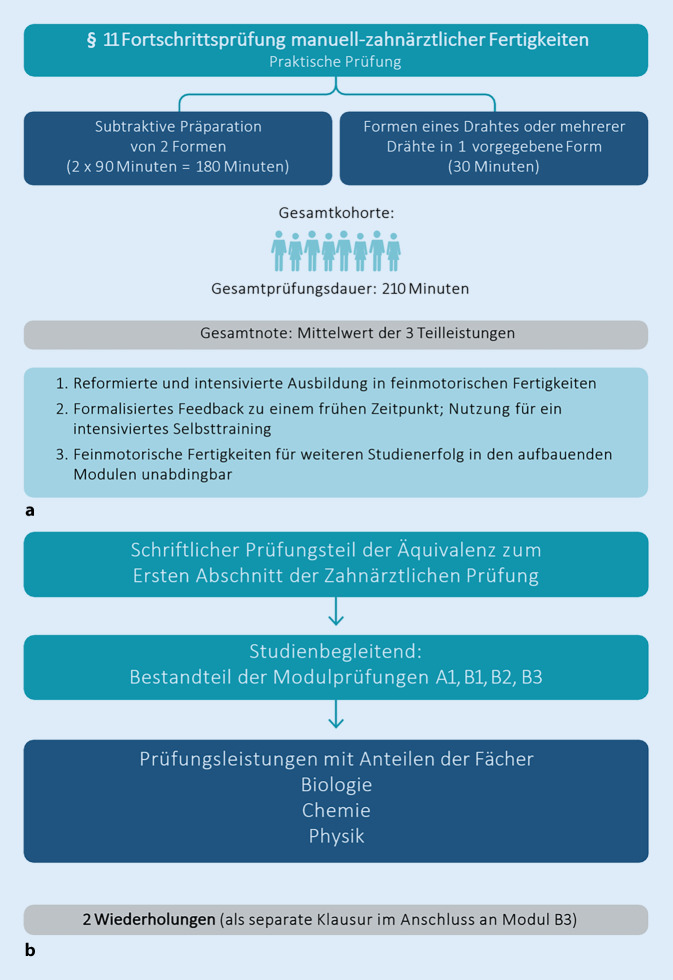

Demselben Ziel dient die Fortschrittsprüfung manuell-zahnärztlicher Fertigkeiten („MFP“) am Ende des ersten Studienjahres (Abb. 2a). Neben der Prüfung relevanten Grundlagenwissens in einem mündlichen Prüfungsteil beinhaltet diese Prüfung einen hoch gewichteten praktischen Prüfungsanteil. In diesem wird anhand grundlegender praktischer Aufgaben (kieferorthopädische Drahtbiegefigur, subtraktive Kauflächengestaltung in der zahnärztlichen Prothetik und geometrische Formenherstellung in der Zahnerhaltung) überprüft, ob die manuelle Voraussetzung für einen weiteren Studienerfolg gegeben ist. Damit der Prüfungszeitpunkt einen engen Bezug zu den gelehrten Inhalten und deren Übung aufweist, findet die MFP bereits nach dem ersten Studienjahr statt und beinhaltet eine summative Beurteilung der erworbenen Fertigkeiten. Studierende erhalten durch das Prüfungsergebnis zu einem frühen Zeitpunkt ein Feedback und können dieses für ein intensiviertes Selbsttraining (Skills Lab, Dentallabor) nutzen. Bei knappem oder Nichtbestehen ist ferner eine frühzeitige Überprüfung der Entscheidung für das Studium der Zahnmedizin möglich.

In iMED DENT ist die Z1-Prüfung durch eine universitäre Äquivalenzprüfung ersetzt (Abb. 2b). Für die Fächer Biologie, Chemie und Physik müssen dafür übergreifend für die 4 Module des ersten Studienjahres jeweils 60 % der jeweiligen Punkte aus den Modulabschlussprüfungen erreicht werden. Für die weiteren Z1-Fächer finden mündliche Prüfungen nach dem vierten Semester statt.

## Herausforderungen und Limitationen der Integration

Vorbild für den integrierten Studiengang iMED DENT waren Modellstudiengänge der Humanmedizin, insbesondere der in Hamburg seit 2012 etablierte Studiengang iMED [1, 2]. In diesem wurden für einzelne Module Leiterkrankungen definiert, die den beteiligten Fächern als Leitlinie für die Integration ihrer Lehre dienen. In der Planungsphase für iMED DENT wurde jedoch deutlich, dass dieses Konzept in strikter Form auf die Zahnmedizin nicht übertragbar ist. Die Gründe dafür liegen in den Besonderheiten des stark praxisorientierten Zahnmedizinstudiums, in dem die Studierenden bereits früh manuelle Fertigkeiten erlernen müssen, die in der Patient:innenbehandlung noch während ihres Studiums zur Anwendung kommen.

Diese häufig das gesamte Modul durchziehenden praktischen Anteile (z. B. die Herstellung von Modellen in Modul B2 oder die Kauflächenpräparationen in Modul B3) bieten nur geringfügig Anknüpfungspunkte, insbesondere für die nicht-zahnmedizinischen Fächer. Konzeptionell schränkt dies die horizontale Integration spezifischer Inhalte (z. B. in Form von Wochenthemen) im Vergleich zur Humanmedizin ein. Hier steht häufig vielmehr eine longitudinale Integration im Fokus des Curriculums. So sind bestimmte Fachanteile als Grundvoraussetzung für zahnmedizinische Kurse zu verstehen, die später im Curriculum verankert sind (z. B. Anatomie, Terminologie, Werkstoffkunde als Voraussetzung für praktische Kurse der Zahnmedizin). Außerdem ist das Spektrum an Erkrankungen oder Symptomen, die sich als Leiterkrankung bzw. Leitsymptom eignen, in der Zahnmedizin deutlich begrenzter als in der Humanmedizin.

Deswegen wurden die Module des ersten Jahres thematisch nicht nach Leiterkrankungen bzw. Leitsymptomen geordnet (dies erfolgt ab dem 2. Studienjahr im Studienabschnitt „Vom Symptom zur Erkrankung“). Vielmehr wurden natürliche Gegebenheiten und Abläufe von zahnmedizinischer Relevanz (z. B. „der Zahn und seine Umgebung“, „Zahn- und Kieferbewegung“) als Leitlinien für die zu integrierenden Fächer gewählt. Als Folge war in einigen Modulen keine unmittelbare und vollumfängliche Integration der zahnmedizinischen und nicht-zahnmedizinischen Fächer möglich. In diesen Bereichen lag der Fokus entsprechend auf einer Integration der nicht-zahnmedizinischen Fächer untereinander und einer Darstellung der longitudinalen Relevanz.

### Curriculare Integration der Fächer

Insbesondere Fächer wie die Kieferorthopädie mussten sich aufgrund der neuen Studienstruktur im Modellstudiengang umorientieren. Die Kieferorthopädie ist ein wichtiges Fach für die Ausbildung der Zahnmediziner:innen, wobei sie ein spezielles Fachgebiet darstellt, für das eine entsprechende dreijährige Weiterbildung zum/r Fachzahnärzt:in absolviert werden muss. Daher sind weitreichende zahnmedizinische Vorkenntnisse zum Verständnis der komplexen Abläufe nötig. Im Regelstudiengang wurde das Fach Kieferorthopädie folglich erst ab dem siebten Semester unterrichtet. Da das Ziel des Modellstudiengangs aber eine Integration der naturwissenschaftlichen Grundlagenfächer in zahnmedizinische Themen und Fächer war, wurde die Kieferorthopädie zu einem longitudinalen Strang ausgeweitet. Aufgrund des frühen Beginns der Vorlesungsveranstaltungen ab dem ersten Semester mussten Lehrinhalte weitreichend angepasst werden, um die Studierenden nicht zu überfordern.

Der frühe kieferorthopädische Einstieg bringt allerdings auch Vorteile mit sich. Im Rahmen des praktischen Kurses im Modul B1 findet die erste Drahtbiegeübung für Studierende statt, die später in der MFP geprüft wird (Abb. 2a). Parallel dazu absolvieren die Studierenden in der Zahnerhaltung Präparationsübungen als erste Vorbereitung auf die späteren Zahnpräparationen an den Patient:innen. Diese Präparationsübungen sind ebenfalls Bestandteil der MFP. Beides ermöglicht den Studierenden, sich frühzeitig mit Materialien vertraut zu machen und ihre manuellen Fähigkeiten zu testen und zu verbessern. Zusätzlich ist die Kieferorthopädie stark mit naturwissenschaftlichen Fächern verknüpft. Insbesondere die Veranstaltungen der Physik werden weitgehend durch Lehrende der Kieferorthopädie unterrichtet. Die Studierenden lernen in den Vorlesungen die Biomechanik anhand von kieferorthopädischen Beispielen kennen und können im Rahmen eines praktischen Kurses selbst Bögen einsetzen und deren Auswirkungen auf die Zähne beobachten. In Modul B2 profitieren die Studierenden ebenfalls durch Veranstaltungen der Kieferorthopädie, in denen in Form des Teamteachings mit der zahnärztlichen Prothetik der gewünschte frühe Patient:innenkontakt zustande kommt. Durch die longitudinale Aufteilung auf das gesamte Studium unterrichten die Lehrenden der Kieferorthopädie im Sinne der Lernspirale und die Studierenden werden so nachhaltig auf ihr umfangreiches Wissen zurückgreifen können.

### Inhaltliche Schwerpunktlegung

Grundsätzlich ist eine erfolgreiche Entwicklung von fachübergreifenden integrativen Aspekten zunächst auf Vorgaben der zahnmedizinischen Fächer angewiesen. Die Abstimmung mit den nicht-zahnmedizinischen Fächern kann sinnvollerweise erst im zweiten Schritt erfolgen. Für die Entwicklung des Abschnitts „Normalfunktion“ wurde diese Diskussion auch durch den NKLZ unterstützt. Die nicht-zahnmedizinischen Fächer haben sich in diesem Prozess im Vergleich zum bisherigen Regelstudiengang weniger stark auf ihre Fachsystematik und mehr auf eine stärkere Integration im Sinne der Relevanz für den zahnmedizinischen Beruf ausgerichtet.

## Fazit

Mit dem Modellstudiengang iMED DENT wurde angestrebt, in den vergangenen Jahren in der Humanmedizin erfolgreich getestete Lehrkonzepte wie integrierte Lehre, früher Patient:innenkontakt und früher Wissenschaftsbezug auf die Zahnmedizin zu übertragen. Für die Grundlagenvermittlung, die im ersten Studienjahr konzentriert und – so weit wie möglich – fächerübergreifend stattfindet, sind die bisherigen Erfahrungen und die Daten der studentischen Evaluationen sehr positiv. Optimierungsmöglichkeiten werden von den beteiligten Lehrenden kontinuierlich verfolgt und umgesetzt. Die Studierenden attestieren dem Studienabschnitt „Normalfunktion“ bereits jetzt, dass er zwar anspruchsvoll und sehr lernintensiv ist, sie sich danach aber gut auf die auf sie zukommenden klinischen Aufgaben vorbereitet fühlen.
